# Finite Bending of Fiber-Reinforced Visco-Hyperelastic Material: Analytical Approach and FEM

**DOI:** 10.3390/ma17010005

**Published:** 2023-12-19

**Authors:** Jafar Pashazadeh, Alireza Ostadrahimi, Mostafa Baghani, Eunsoo Choi

**Affiliations:** 1 School of Mechanical Engineering, Collage of Engineering, University of Tehran, Tehran 14155-6455, Iran; jafar.pashazadeh@ut.ac.ir; 2 Department of Mechanical and Industrial Engineering, Louisiana State University, Baton Rouge, LA 70803, USA; 3 Department of Civil and Environmental Engineering, Hongik University, Seoul 04066, Republic of Korea; eunsoochoi@hongik.ac.kr

**Keywords:** anisotropic visco-hyperelasticity, fiber-reinforced materials, constitutive model, finite element analysis, relaxation tests

## Abstract

This paper presents a new anisotropic visco-hyperelastic constitutive model for finite bending of an incompressible rectangular elastomeric material. The proposed approach is based on the Mooney–Rivlin anisotropic strain energy function and non-linear visco-hyperelastic method. In this study, we aim to examine the mechanical response of a reinforced viscoelastic rectangular bar with a group of fibers under bending. Anisotropic materials are typically composed of one (or more) family of reinforcing fibers embedded within a soft matrix material. This operation may lead to an enhancement in the strength and stiffness of soft materials. In addition, a finite element simulation is carried out to validate the accuracy of the analytical solution. In this research, the well-known stress relaxation test, as well as the multi-step relaxation test, are examined both analytically and numerically. The results obtained from the analytical solution are found to be in good agreement with those from the finite element method. Therefore, it can be deduced that the proposed model is competent in describing the mechanical behavior of fiber-reinforced materials when subjected to finite bending deformations.

## 1. Introduction

Soft fiber-reinforced composites are widely used in both industrial and biological applications including automotive, damping structures, blood vessels, and ligaments. These materials are characterized by their specific strength-to-density ratio, energy absorption capability, flexibility, and electrochemical storage devices [1,2,3,4]. A soft fiber-reinforced material may exhibit a wide range of non-linear behaviors such as anisotropy [5], viscoelasticity [6], temperature-dependency [7], and damage phenomenon [8]. The general anisotropic property is caused by a number of fibers that are organized into a soft polymeric matrix called ground substance [9,10]. Furthermore, most soft materials in nature, such as soft biological tissues, can also possess anisotropic properties due to the reinforcing fibers [11,12]. The simplest form of anisotropy can be achieved by the reinforcement of a soft material with only one family of fibers. Consequently, it is important to note that the direction of fibers has a significant role in the mechanical behavior of anisotropic materials.

In the last few years, researchers have been focused mainly on the viscoelastic behavior of anisotropic soft materials. The mechanical response of viscoelastic materials generally depends on the rate of deformation, which can be described by rheological models consisting of elastic springs and viscous dashpots [13,14,15]. Accordingly, Holzapfel and Gasser [9] introduced a fully three-dimensional model for anisotropic viscoelastic composites based on evolution equations for two families of fibers. Diani, Brieu, and Gilormini [16] proposed an analytical viscoelastic model for anisotropic rubber-like materials under cyclic uniaxial tension. Nguyen, Jones, and Boyce [17] presented an anisotropic viscoelastic solution for soft fiber reinforced composites, using the distinct decomposition of deformation gradient tensors into the matrix and fiber parts. Rajagopal and Wineman [18] investigated the non-linear behavior of anisotropic viscoelastic solids for different deformation regimes such as biaxial, triaxial, shear, and extension–torsion deformations. Kulkarni, Gao, Horner, Mortlock, and Zheng [19] expressed a transversely isotropic visco-hyperelastic constitutive model for soft tissues, like porcine and human brain tissues, under large deformations and high strain rates. Anani and Rahimi [20] proposed a constitutive model to describe the visco-hyperelastic behavior of transversely isotropic functionally graded rubbers at different strain rates. Yang, Yao, Yan, Yuan, Dong, and Liu [21] represented an anisotropic visco-hyperelastic model, considering the effects of temperature on fiber-reinforced rubber composites. Additionally, Fard and Vakili-Tahami [22] developed a coupled thermo-visco-hyperelastic damage model for soft fiber-reinforced tissues based on a thermodynamic framework.

In material engineering science, researchers have always been interested in the mechanical behavior of hyperelastic materials under different deformation regimes. Recently, the classical subject of finite bending of rectangular elastic materials has been widely investigated due to its various applications. This problem was first discussed by Rivlin in 1949 for general highly elastic incompressible rectangular systems [23,24]. In a subsequent study, Taber [25] examined the bending problem within the context of embryonic cardiac loops. Kanner and Horgan [26] introduced an analytical model to express the plane strain bending of an incompressible isotropic elastic material subjected to an end moment. Later, Shojaeifard, Sheikhi, Baniassadi, and Baghani [27] suggested a constitutive framework to represent the time-dependent behavior of visco-hyperelastic rectangular materials under finite bending. In our previous work, a new model was proposed for the stress-softening of an incompressible hyperelastic rectangular bar under cyclic bending and unbending deformation [28]. In addition, Bakhtiyari, Baniasadi, and Baghani [29] proposed a thermo-visco-hyperelastic approach for shape memory polymers under large bending deformations and temperature changes.

In the present study, we developed an analytical solution for describing the time-dependent behavior of anisotropic, incompressible, visco-hyperelastic rectangular materials under finite bending deformation. For this purpose, a separable constitutive model is introduced based on the Mooney–Rivlin anisotropic strain energy function [9,30,31] and non-linear visco-hyperelastic theory [32]. It should be noted that anisotropic strain energy functions are typically composed of two parts. One part relates to the stored energy in the matrix material, while the second part refers to the stored energy within the fibers. As compared to previous studies on the bending problem, a key innovation of this paper is the anisotropy of the viscoelastic material. In this work, a viscoelastic rubber-like material is used as the matrix, which is reinforced by one family of fibers. In order to study the influence of fiber direction, we evaluated different orientations of fibers embedded in the matrix. As part of this work, a finite element analysis has also been performed to verify the accuracy of the proposed analytical approach. 

This paper is organized as follows: In Section 2, the governing equations and the solution method are discussed. Section 3 describes the finite element analysis and numerical implementation of the problem. In Section 4, the results obtained through analytical methods are discussed and then compared with those of FEM. As a final step, a summary and some conclusions are presented in the last section.

## 2. Governing Equations

### 2.1. Basic Kinematics of Anisotropic Hyperelastic Materials

**Figure 1:**
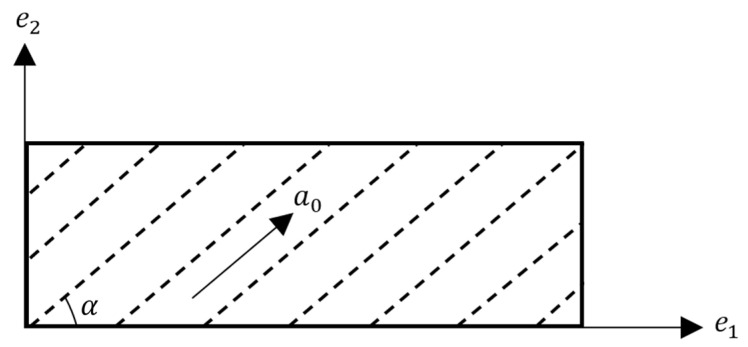
Schematic of fiber direction with the unit vector of $a_{0}$.

Let X and x determine the position vectors in the reference and current configurations, respectively. In continuum mechanics, the deformation gradient tensor is defined by F=∂x/∂X, which is mapping the vector dX from the reference configuration to the vector of dx in the current configuration. The right and left Cauchy–Green deformation tensors are given by $C=F^{T}F$ and $B=FF^{T}$, respectively, where the superscript T denotes the transpose of a tensor. For an isotropic hyperelastic material, we need three principal invariants of C, as follows [19,33]:

(1) $$\begin{array}{c} I_{1}=trC=\lambda_{1}^{2}+\lambda_{2}^{2}+\lambda_{3}^{2},\\ I_{2}=\frac{1}{2}[(trC{)}^{2}-tr(C^{2})]=\lambda_{1}^{2}\lambda_{2}^{2}+\lambda_{2}^{2}\lambda_{3}^{2}+\lambda_{1}^{2}\lambda_{3}^{2},\\ I_{3}=\det\;C=\lambda_{1}^{2}\lambda_{2}^{2}\lambda_{3}^{2}. \end{array}$$

where $\lambda_{i}$ (i=1,2,3) stands for the principal stretches of C. In addition, for an anisotropic hyperelastic material with one family of reinforcing fibers, two additional invariants are given as follows [19]:

(2) $$I_{4}=a_{0}.Ca_{0},\;\;\;\;\;I_{5}=a_{0}.C^{2}a_{0}$$

where $a_{0}$ is a unit vector in the reference configuration which describes the direction of the family of fibers. It is worth noting that $I_{5}$ is a pseudo-invariant of C. For a fiber-reinforced plane strain material, the direction of fibers can be determined by the following:

(3) $$a_{0}=\left(\begin{array}{c} \cos\;\alpha \\ \sin\;\alpha \\ 0 \end{array}\right)$$

where α denotes the initial angle between the fiber direction and the first axis of reference configuration, $e_{1}$, as shown in Figure 1. In the same way, it is possible to define two additional invariants ($I_{6}$, $I_{7}$) with corresponding unit vectors for materials consisting of two families of fibers.

In continuum mechanics, the mechanical properties of anisotropic hyperelastic materials can be determined by a decoupled strain energy density function, Ψ, per unit of undeformed volume. It may be defined either by the principal stretches or the left Cauchy–Green tensor invariants. In terms of the invariants, Ψ can be written as follows [34]:

(4) $$\Psi \left(C,a_{0}\right)=\Psi_{vol}\left(\;J\right)+\Psi_{iso}\left(C,a_{0}\right)=\Psi_{vol}\left(\;J\right)+\Psi_{iso}\left(I_{1},I_{2},I_{4},I_{5}\right).$$

where $\Psi_{vol}$ indicates the strain energy associated with volume changes and J is the determinant of the deformation gradient tensor F. On the other hand, $\Psi_{iso}$ is regarded as the isochoric part of the strain energy, which can be decomposed into the isotropic and anisotropic contributions due to the matrix and fibers. It should be noted that for incompressible materials, we can write det F=1 or $I_{3}=J=1$.

In order to obtain the constitutive equations for an incompressible anisotropic hyperelastic material, the second Piola–Kirchhoff stress tensor is defined as follows [35]:

(5) $$\begin{array}{c} S=2\frac{\partial \Psi \left(C,a_{0}\right)}{\partial C}\\ \;=2\left[\left(\frac{\partial \Psi }{\partial I_{1}}+I_{1}\frac{\partial \Psi }{\partial I_{2}}\right)I-\frac{\partial \Psi }{\partial I_{2}}C+\frac{\partial \Psi }{\partial I_{4}}\left(a_{0}⊗a_{0}\right)+\frac{\partial \Psi }{\partial I_{5}}\left(a_{0}⊗Ca_{0}+Ca_{0}⊗a_{0}\right)\right]-pC^{-1}. \end{array}$$

where p shows the hydrostatic pressure and I refers to the identity matrix. Moreover, the Cauchy stress tensor may be found by transferring the second Piola–Kirchhoff stress tensor to the current configuration, according to $\sigma =J^{-1}FSF^{T}$. Therefore, using Equation (5), one can compute the strain-dependent Cauchy stress tensor for anisotropic hyperelastic materials via the following equation [36]:

(6) $$\sigma =2\left[\left(\frac{\partial \Psi }{\partial I_{1}}+I_{1}\frac{\partial \Psi }{\partial I_{2}}\right)B-\frac{\partial \Psi }{\partial I_{2}}B^{2}+\frac{\partial \Psi }{\partial I_{4}}\left(a⊗a\right)+\frac{\partial \Psi }{\partial I_{5}}\left(a⊗Ba+Ba⊗a\right)\right]-pI.$$

where $a=Fa_{0}$ describes the relation between the fiber direction in the reference and current configurations.

### 2.2. Anisotropic Visco-Hyperelastic Model

To describe the viscoelastic effect, the finite strain anisotropic viscoelastic model proposed in Holzapfel [32] is considered. A visco-hyperelastic material can be expressed via rheological models that incorporate elastic springs and viscous dashpots. Utilizing the generalized Maxwell model, the strain energy density function can be rewritten as follows [37,38]:

(7) $$\Psi \left(C,a_{0},\Gamma_{1},\ldots ,\Gamma_{m}\right)=\Psi_{vol}^{\infty }\left(\;J\right)+\Psi_{iso}^{\infty }\left(C,a_{0}\right)+\sum_{i=1}^{m}\gamma_{i}\left(C,\Gamma_{i}\right).$$

where $\Psi_{vol}^{\infty }$ and $\Psi_{iso}^{\infty }$ are the volumetric and isochoric part of the strain energy function and characterize the equilibrium state of the materials in which the superscript ∞ determines the long-term equilibrium. The additional term of $\sum_{i=1}^{m}\gamma_{i}$ shows the power dissipation and relates for the viscoelastic response. The total response of the second Piola–Kirchhoff stress is computed in the form of the following equation [9]:

(8) $$S=2\frac{\partial \Psi \left(C,a_{0},\Gamma_{1},\ldots ,\Gamma_{m}\right)}{\partial C}=S_{vol}^{\infty }+S_{iso},$$

with the definition

(9) $$S_{iso}=S_{iso}^{\infty }+\sum_{i=1}^{m}Q_{i},$$

where $Q_{i}$ refers to the isochoric non-equilibrium stress tensor defined by the following:

(10) $$Q_{i}=2\frac{\partial \gamma_{i}\left(C,\Gamma_{i}\right)}{\partial C}.$$

The next step is to formulate the evolution equations for each branch of viscous damping. The isochoric non-equilibrium stress tensor, $Q_{i}$, is assumed to be obtained by the following:

(11) $${\dot{Q}}_{i}+\frac{Q_{i}}{\tau_{i}}={\dot{S}}_{iso,i}.$$

where $\tau_{i}\in \left(0,\infty \right)$ is the material parameter and represents the relaxation time. $S_{iso,i}$ defines the isochoric part of the second Piola–Kirchhoff stress tensor corresponding to the strain energy $\Psi_{iso,i}^{\infty }\left(C,a_{0}\right)$ of the material and could be derived as follows:

(12) $$\Psi_{iso,i}^{\infty }\left(C,a_{0}\right)=\beta_{i}\Psi_{iso}^{\infty }\left(C,a_{0}\right)\;\Rightarrow \;S_{iso,i}=2\frac{\partial \Psi_{iso,i}^{\infty }\left(C,a_{0}\right)}{\partial C}=2\beta_{i}\frac{\partial \Psi_{iso}^{\infty }\left(C,a_{0}\right)}{\partial C}={\beta_{i}S}_{iso}^{\infty }.$$

where $\beta_{i}\in \left(0,\infty \right)$ stands for the energy factors and $S_{iso}^{\infty }$ represents the strain-dependent or hyperelastic response of the material.

Finding the total viscoelastic stress S and the non-equilibrium stresses $Q_{i}$ from Equation (11) may be expressed by a convolution integral in the form of the following [38]:

(13) $$Q_{i}=\int_{t=0}^{t=T}\exp\;\left(-\frac{T-t}{\tau_{i}}\;\right)\frac{dS_{iso,i}(t)}{dt}dt.$$

In order to obtain the updated algorithm of the second Piola–Kirchhoff stress S, we must consider a time discretization $∆t=t_{n+1}-t_{n}$ of the time interval $\left[0,T\right]$. The Equation (13) can be recast as follows:

(14) $$\begin{array}{c} Q_{i,n+1}=\int_{0}^{t_{n}}\exp\;\left(-\frac{∆t+t_{n}-t}{\tau_{i}}\;\right)\frac{dS_{iso,i}\left(t\right)}{dt}dt+\int_{t_{n}}^{t_{n+1}}\exp\;\left(-\frac{t_{n+1}-t}{\tau_{i}}\;\right)\frac{dS_{iso,i}\left(t\right)}{dt}dt\\ =\exp\;\left(-\frac{∆t}{\tau_{i}}\;\right)Q_{i,n}+\int_{t_{n}}^{t_{n+1}}\exp\;\left(-\frac{t_{n+1}-t}{\tau_{i}}\;\right)\frac{dS_{iso,i}\left(t\right)}{dt}dt. \end{array}$$

Utilizing Equations (12) and (14), the total viscoelastic stress at the time step of $t_{n+1}$ could be calculated as follows:

(15) $$\begin{array}{c} S_{n+1}={\left.\left(S_{vol}^{\infty }+S_{iso}^{\infty }+\sum_{i=1}^{m}Q_{i}\right)\right|}_{n+1}\\ =S_{vol}^{\infty }+S_{iso}^{\infty }+\sum_{i=1}^{m}exp\left(-\frac{∆t}{\tau_{i}}\right)Q_{i,n}+exp\left(-\frac{∆t}{{2\tau }_{i}}\right)\beta_{i}\left(S_{iso,n+1}^{\infty }-S_{iso,n}^{\infty }\right), \end{array}$$

with the following definitions [32]:

(16) $$S_{vol,n+1}^{\infty }=J_{n+1}\frac{\partial \Psi_{vol}^{\infty }\left(\;J_{n+1}\right)}{\partial J_{n+1}}C_{n+1}^{-1},\;\;\;\;\;\;\;\;\;S_{iso,n+1}^{\infty }=2\frac{\partial \Psi_{iso}^{\infty }\left(C_{n+1},a_{0}\right)}{\partial C_{n+1}}.$$

According to Equation (15), the strain-dependent or hyperelastic response of the material should be calculated at each time increment. In the following, a framework for fiber-reinforced hyperelastic rectangular materials undergoing finite bending deformation is presented.

### 2.3. Finite Bending of Anisotropic Hyperelastic Materials

In this work, the fiber-reinforced material is assumed to be incompressible, which ideally means that the volume will not change during the deformation (det F=1 or $I_{3}=J=1$). Therefore, the volumetric part of the total stress, $S_{vol}^{\infty }$, can be ignored. In this section, we will determine the isochoric response of the second Piola–Kirchhoff stress, $S_{iso}^{\infty }$, under pure bending. 

The common Mooney–Rivlin strain energy function for anisotropic hyperelastic materials is as follows [9,39]:

(17) $$\Psi_{iso}^{\infty }\left(I_{1},I_{2},I_{4},I_{6}\right)=c_{1}\left(I_{1}-3\right)+c_{2}\left(I_{2}-3\right)+\frac{k_{1}}{2k_{2}}\left\{\exp\;\left[k_{2}{\left(I_{4}-1\right)}^{2}\right]-1\right\},$$

which can be decomposed into two parts as follows:

(18) $$\Psi_{iso\left(isotropic\right)}^{\infty }=c_{1}\left(I_{1}-3\right)+c_{2}\left(I_{2}-3\right),\;\;\;\;\;\;\;\Psi_{iso\left(anisotropic\right)}^{\infty }=\frac{k_{1}}{2k_{2}}\left\{\exp\;\left[k_{2}{\left(I_{4}-1\right)}^{2}\right]-1\right\}.$$

where $\Psi_{iso\left(isotropic\right)}^{\infty }$ and $\Psi_{iso\left(anisotropic\right)}^{\infty }$ depict the isotropic and anisotropic parts of the strain energy function associated with the matrix and fibers, respectively. $I_{1}$ and $I_{2}$ are the first and second deformation invariants, and $I_{4}$ is the fourth deformation pseudo-invariant. Also, $c_{1}$ and $c_{2}$ illustrate the matrix material parameters, and $k_{1}$ and $k_{2}$ refer to the fiber stiffness and fiber non-linearity, respectively.

**Figure 2:**
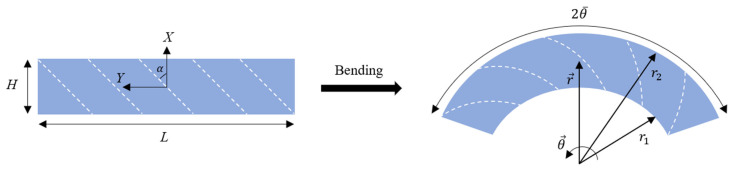
Bending of a fiber-reinforced visco-hyperelastic rectangular bar with an initial fiber angle of α.

In order to achieve the anisotropic hyperelastic solution, we need to configure the kinematics of the bending process. As shown in Figure 2, the reference and current coordinates are considered to be cartesian (X,Y,Z) and cylindrical (r,θ,z), respectively.

Regarding Figure 2, the term $2\bar{\theta }$ defines the ultimate bending angle of the bar. As shown in Figure 2, the main directions X and Y are oriented in the direction of the thickness and length of the bar, respectively, which have a 90 degrees counter-clockwise rotation when compared to the main directions in Figure 1. Since the deformations generated in fiber-reinforced anisotropic materials are of a similar type, it is possible to assume that the entire structure of the anisotropic rectangular material will exhibit a deformation field as follows [26,40]:

(19) $$r=f\left(X,t\right),\;\;\theta =\frac{Y}{\rho \left(t\right)},\;\;z=Z\;with\left\{\begin{array}{c} -H/2\leq X\leq H/2,\\ -\frac{L}{2}\leq Y\leq \frac{L}{2},\\ -\infty \leq Z\leq +\infty . \end{array}\right.$$

where $1/\rho \left(t\right)$ stands for the curvature of the bar and H and L show the width and length of the bar, respectively. Employing Equation (19), the deformation gradient tensor becomes as follows:

(20) $$F=\left[\begin{array}{ccc} \frac{df(X,t)}{dY} & 0 & 0\\ 0 & \frac{f(X,t)}{\rho (t)} & 0\\ 0 & 0 & 1 \end{array}\right].$$

Considering the incompressibility constraint, det F=1, an ordinary differential equation will be derived, and by solving that equation, f(X,t) will be acquired. As a result, f(X,t) will become a function that represents radius changes over time, as follows:

(21) $$f\left(X,t\right)=r\left(t\right)=\sqrt{2\rho \left(t\right)X+c\left(t\right)}.$$

where c(t) is the integration constant. Moreover, the inner and outer radii will be determined by replacing −H/2 and +H/2 instead of X in Equation (21), respectively:

(22) $$r_{1}\left(t\right)=\sqrt{c\left(t\right)-\rho \left(t\right)H}\;,\;\;\;\;\;\;\;\;r_{2}\left(t\right)=\sqrt{c\left(t\right)+\rho \left(t\right)H}.$$

With the help of Equations (20) and (21), as well as employing Equations (1) and (2), the left (and right) Cauchy–Green deformation tensor and its invariants can be obtained as follows:

(23) $$B\left(=C\right)=\left[\begin{array}{ccc} \frac{\rho (t{)}^{2}}{r(t{)}^{2}} & 0 & 0\\ 0 & \frac{r(t{)}^{2}}{\rho (t{)}^{2}} & 0\\ 0 & 0 & 1 \end{array}\right],$$

where:

(24) $$\begin{array}{c} I_{1}=I_{2}=\frac{\rho (t{)}^{2}}{r(t{)}^{2}}+\frac{r(t{)}^{2}}{\rho (t{)}^{2}}+1,\;\;\;\;\;\;\;I_{3}=1,\\ I_{4}=\frac{\rho (t{)}^{2}}{r(t{)}^{2}}{cos}^{2}\alpha +\frac{r(t{)}^{2}}{\rho (t{)}^{2}}{sin}^{2}\alpha ,\;\;\;\;\;\;\;I_{5}=\frac{\rho (t{)}^{4}}{r(t{)}^{4}}{cos}^{2}\alpha +\frac{r(t{)}^{4}}{\rho (t{)}^{4}}{sin}^{2}\alpha . \end{array}$$

Utilizing the Cauchy stress equation, Equation (6), the Mooney–Rivlin anisotropic strain energy function, and Equation (17), the strain-dependent stress components can be obtained by the following:

(25) $$\begin{array}{c} \sigma_{rr}^{\infty }=-p\left(t\right)+2\frac{\rho (t{)}^{2}}{r(t{)}^{2}}\frac{\partial \Psi_{iso}^{\infty }}{\partial I_{1}}-2\frac{r(t{)}^{2}}{\rho (t{)}^{2}}\frac{\partial \Psi_{iso}^{\infty }}{\partial I_{2}}+2\frac{\rho (t{)}^{2}}{r(t{)}^{2}}{cos}^{2}\alpha \frac{\partial \Psi_{iso}^{\infty }}{\partial I_{4}},\\ \sigma_{\theta \theta }^{\infty }=-p\left(t\right)+2\frac{r(t{)}^{2}}{\rho (t{)}^{2}}\frac{\partial \Psi_{iso}^{\infty }}{\partial I_{1}}-2\frac{\rho (t{)}^{2}}{r(t{)}^{2}}\frac{\partial \Psi_{iso}^{\infty }}{\partial I_{2}}+2\frac{r(t{)}^{2}}{\rho (t{)}^{2}}{sin}^{2}\alpha \frac{\partial \Psi_{iso}^{\infty }}{\partial I_{4}},\\ \sigma_{zz}^{\infty }=-p\left(t\right)+2\frac{\partial \Psi_{iso}^{\infty }}{\partial I_{1}}-2\frac{\partial \Psi_{iso}^{\infty }}{\partial I_{2}},\\ \sigma_{r\theta }^{\infty }=\frac{\partial \Psi_{iso}^{\infty }}{\partial I_{4}}\sin\;2\alpha . \end{array}$$

where the shear stress $\sigma_{r\theta }^{\infty }$ is generated by the presence of reinforcing fibers. 

In the absence of body forces, the simplified form of the first equilibrium equation in the current cylindrical configuration is as follows [28]:

(26) $$\frac{\partial \sigma_{rr}^{\infty }}{\partial r}+\frac{1}{r}(\sigma_{rr}^{\infty }-\sigma_{\theta \theta }^{\infty })=0.$$

Next, by differentiating the strain energy function with respect to the radius, and employing the left (or right) Cauchy–Green tensor invariants, we have the following:

(27) $$\begin{array}{c} \frac{d\Psi_{iso}^{\infty }}{dr}=\frac{\partial \Psi_{iso}^{\infty }}{\partial I_{1}}\frac{\partial I_{1}}{\partial r}+\frac{\partial \Psi_{iso}^{\infty }}{\partial I_{2}}\frac{\partial I_{2}}{\partial r}+\frac{\partial \Psi_{iso}^{\infty }}{\partial I_{4}}\frac{\partial I_{4}}{\partial r}\\ =2\left(\frac{r(t)}{\rho (t{)}^{2}}-\frac{\rho (t{)}^{2}}{r(t{)}^{3}}\right)\left[\frac{\partial \Psi_{iso}^{\infty }}{\partial I_{1}}+\frac{\partial \Psi_{iso}^{\infty }}{\partial I_{2}}\;\right]+\left(\frac{r(t)}{\rho (t{)}^{2}}{sin}^{2}\alpha -\frac{\rho (t{)}^{2}}{r(t{)}^{3}}{cos}^{2}\alpha \right)\frac{\partial \Psi_{iso}^{\infty }}{\partial I_{4}}=-\frac{1}{r}\left(\sigma_{rr}^{\infty }-\sigma_{\theta \theta }^{\infty }\right). \end{array}$$

In light of Equations (26) and (27), one may obtain the radial stress as follows:

(28) $$\;\;\frac{d\Psi_{iso}^{\infty }}{dr}=\frac{\partial \sigma_{rr}^{\infty }}{\partial r}\;\;\;\Rightarrow \;\;\;\sigma_{rr}^{\infty }\left(r\right)=\Psi_{iso}^{\infty }\left(r\right)+K,$$

where K is the integration constant. Applying the free-stress surfaces boundary conditions, K is calculated as follows:

(29) $$\sigma_{rr}^{\infty }\left(r_{1}\right)=\sigma_{rr}^{\infty }\left(r_{2}\right)=0\;\;\;\;\Rightarrow \;\;\;\;K=-\Psi_{iso}^{\infty }\left(r_{1}\right)=-\Psi_{iso}^{\infty }\left(r_{2}\right).$$

Using the above result, one may find the hydrostatic pressure p(t). In this regard, employing the radial stress from Equation (25) and the relations (28) and (29), we have the following:

(30) $$p\left(t\right)=2\frac{\rho (t{)}^{2}}{r(t{)}^{2}}\frac{\partial \Psi_{iso}^{\infty }}{\partial I_{1}}-2\frac{r(t{)}^{2}}{\rho (t{)}^{2}}\frac{\partial \Psi_{iso}^{\infty }}{\partial I_{2}}+2\frac{\rho (t{)}^{2}}{r(t{)}^{2}}{cos}^{2}\alpha \frac{\partial \Psi_{iso}^{\infty }}{\partial I_{4}}+\Psi_{iso}^{\infty }\left(r_{1}\right)-\Psi_{iso}^{\infty }\left(r\right).$$

During the bending deformation, the radius of curvature $\rho \left(t\right)$ for incompressible materials is related to both the inner and outer radii as follows [24]:

(31) $$\rho \left(t\right)=\sqrt{r_{1}\left(t\right)\;r_{2}\left(t\right)}.$$

Substituting $r_{1}(t)$ and $r_{2}(t)$ from Equation (22), one may find the integration constant $c\left(t\right)$ as follows:

(32) $$c\left(t\right)=\rho \left(t\right)\sqrt{\rho (t{)}^{2}+H^{2}}.$$

Furthermore, the resultant bending moment and resultant normal force can be derived by using the total tangential stress through the following equations, respectively:

(33) $$M(t)=\int_{r_{1}}^{r_{2}}r(t)\sigma_{\theta \theta }(t)dr$$

(34) $$N(t)=\int_{r_{1}}^{r_{2}}\sigma_{\theta \theta }(t)dr$$

## 3. Finite Element Method (FEM)

**Figure 3:**
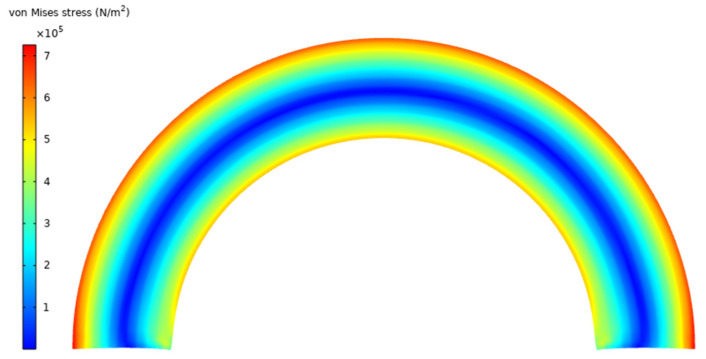
Bending of anisotropic visco-hyperelastic rectangular bar using FEM.

In this section, a finite element analysis is carried out for the bending of an incompressible anisotropic visco-hyperelastic rectangular bar composed of a polymeric matrix substance and one family of reinforcing fibers (see Figure 3). In order to achieve this, the COMSOL Multiphysics 6.0 software package was used to simulate the current problem. The anisotropic type of Mooney–Rivlin strain energy function and non-linear visco-hyperelastic method are utilized to define the strain-dependent and time-dependent behaviors of the fiber-reinforced bar, respectively. A rectangular 2D plane strain deformable solid is chosen with a length of 0.8 m and a height of 0.1 m. To precisely analyze the mechanical properties of the anisotropic bar, the fibers are oriented at angles ranging from α=0° to α=90° in the reference configuration. Also, the bar is bent up to the maximum bending angle of $2\bar{\theta }=180{}^{\circ}$. In Figure 2, we showed how the fibers also undergo bending deformation. A 4-node plane strain quadrilateral element type is used to mesh the structure. Further, mesh independency was examined to ensure the convergence of the simulation. The model consists of 3500 domain elements, which are sufficient for the current problem. The material properties are listed in Table 1.

## 4. Results and Discussion

This section presents the results obtained from the analytical method and compares them with the FEM results to verify the accuracy of the proposed approach. In the following, two standard deformation modes, the stress relaxation test and multi-step relaxation test, are studied in detail for different fiber angles. It should be noted that in all tests, the anisotropic bar has a length of 0.8 m and a height of 0.1 m.

In this problem, the inclusion of fibers in the matrix material leads to a slight shear stress. Equation (25) indicates that the shear stress is zero when the fiber angles are α=0° and α=90°. Hence, both analytical and numerical results are consistent with these fiber angles. However, if the fibers are inserted into the matrix material at angles ranging from 0° to 90°, a small discrepancy may arise due to the presence of shear stress.

### 4.1. Stress Relaxation Test

**Figure 4:**
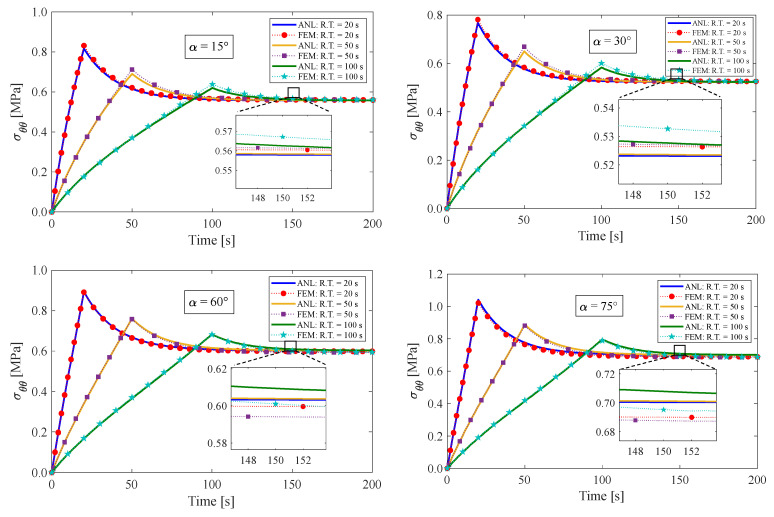
Variations of tangential stress in the outer radius of the bent bar for different fiber angles of α. The ultimate bending angle is specified to be 180°.

**Figure 5:**
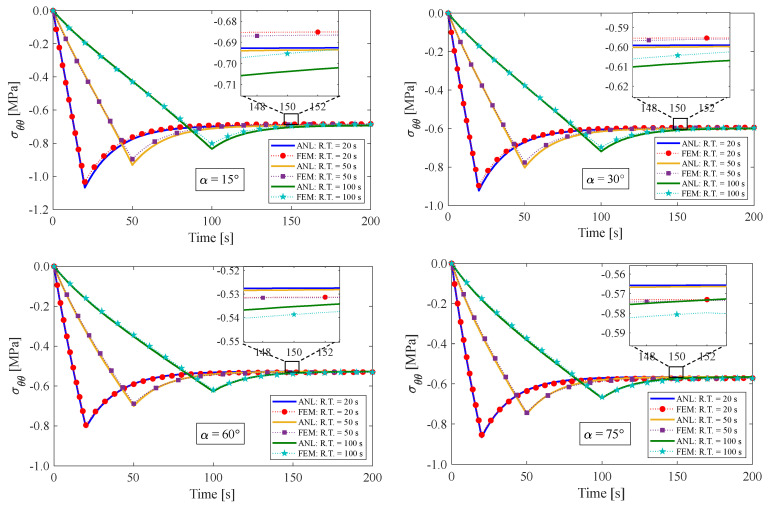
Variations of tangential stress in the inner radius of the bent bar for different fiber angles of α. The ultimate bending angle is determined to be 180°.

This section presents the response of the fiber-reinforced visco-hyperelastic model to a finite bending relaxation test under various conditions. The fibers are embedded into the rectangular matrix material with different initial angles. Initially, the anisotropic rectangular material is bent until it reaches a maximum bending angle (B.A.) of 180° with three different rising times (R.T.) of 20 s, 50 s, and 100 s, and then, the bending angle is kept fixed for a period of time. Figure 4 and Figure 5 depict the changes in tangential stress in the outer and inner surfaces of the material, respectively. It can be observed that the stress components rise up to their maximum values during the rising time and then gradually decrease until they reach equilibrium values. Figure 4 shows that the outer radius experiences tensile stress due to tensile loads applied. On the other hand, as expected, the tangential stress at the inner radius of the bent bar is compressive (see Figure 5). As mentioned earlier, the material is reinforced by a family of fibers with the same initial angle of α. It can be noted that by increasing the fiber angle from α=0° to α=45°, the magnitude of the stress components decreases; however, by further increasing the fiber angle up to α=90°, the value of the stress components increases. It could be said that this point is the result of a change in the tensile strength of the material for different fiber angles. As mentioned, the presence of fibers in the matrix material with angles between 0° and 90° causes shear stresses under bending, which leads to a small error in the response of the material. We can point out as a limitation of the presented approach that choosing fibers from softer materials compared to the matrix can reduce the model error. Table 2 displays the error percentage between the analytical and numerical results for different fiber angles. The meaning of error in this part can be summed up as the biggest difference between the obtained analytical and numerical answers. Based on these findings, it can be concluded that the analytical approach proposed in this paper can effectively predict the mechanical response of anisotropic materials under finite bending deformations.

**Figure 6:**
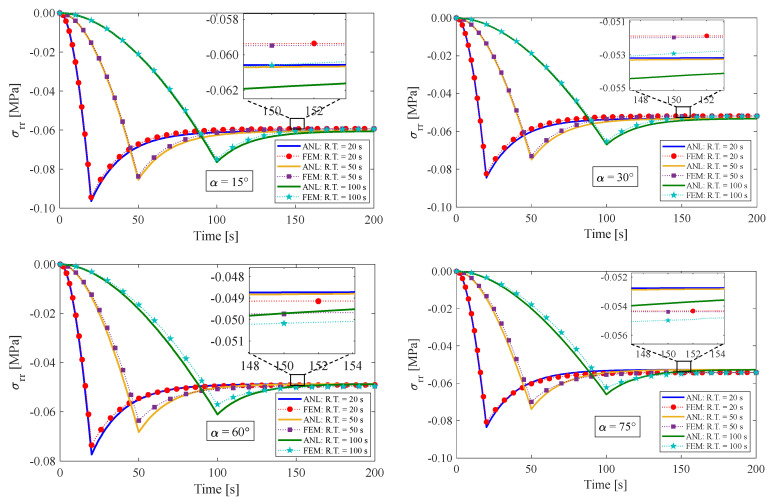
Variations of radial stress in the neutral axis of the bent bar for different fiber angles of α. The ultimate bending angle is defined as 180°.

**Figure 7:**
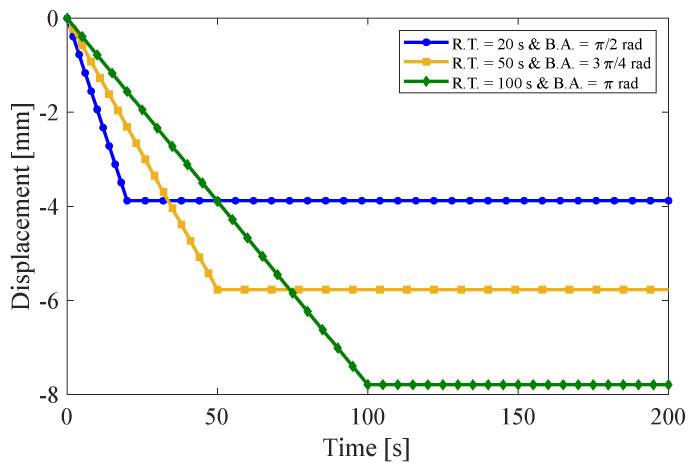
Displacement of the neutral axis for the ultimate bending angles of 90°, 135°, and 180°.

Figure 6 represents the variation of radial stress along the neutral axis of the bent bar for different fiber angles. It is important to note that the inner and outer surfaces of the bent bar have zero radial stress, while the maximum value is observed on the neutral axis. The neutral axis refers to a longitudinal axis in the middle section of the bar, where there is no stretch during bending. The findings from Figure 6 indicate that the radial stresses on the bent bar are compressive. Due to the incompressibility of the fiber-reinforced material, the neutral axis has a displacement toward the inner surface through bending deformation. Figure 7 illustrates the magnitude of displacement of the neutral axis for the ultimate bending angles of 90°, 135°, and 180° at different arbitrary rising times.

**Figure 8:**
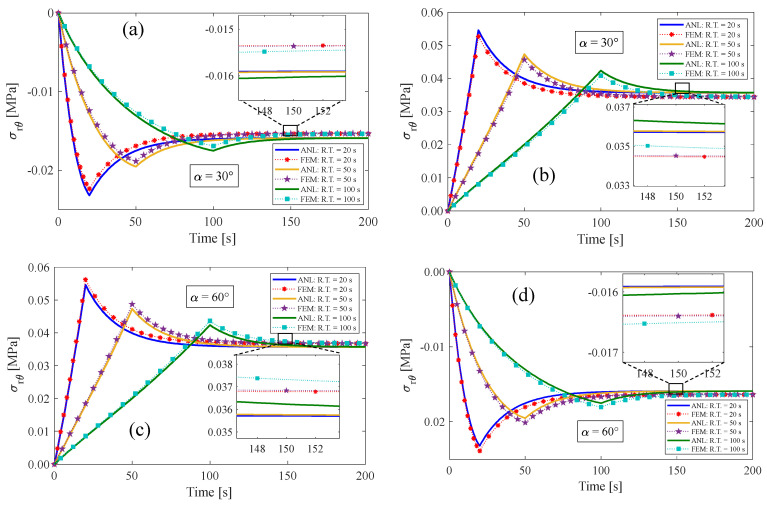
Variation of shear stress on the (**a**,**c**) outer radius and (**b**,**d**) inner radius of the bent bar for two fiber angles of $\alpha =30^{{}^{\circ}},\;60^{{}^{\circ}}$. The ultimate bending angle is determined to be 180°.

Figure 8 presents the alterations in shear stress caused by the inclusion of fibers, which aids in comprehending the material’s behavior. The results obtained suggest that when the sum of two fiber angles is 90°, the shear stress in the inner radius closely approximates the shear stress in the outer radius. It can be argued that for fiber angles ranging from 0 to 45 degrees, the shear stress is negative at the outer radius and positive at the inner radius. However, a reverse effect is observed for fiber angles between 45 and 90 degrees. Furthermore, it is found that there is no significant shear stress near by the neutral axis. Since the shear stress within the material is zero at fiber angles of 0 and 90 degrees, both analytical and numerical responses are similar, showing a good accuracy. However, for the fiber angles ranging from 0 to 90 degrees, the bending of a fiber-reinforced material causes shear stress. This leads to a slight discrepancy between the two solution methods.

**Figure 9:**
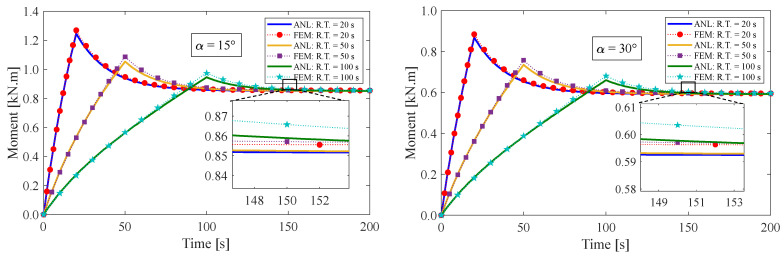

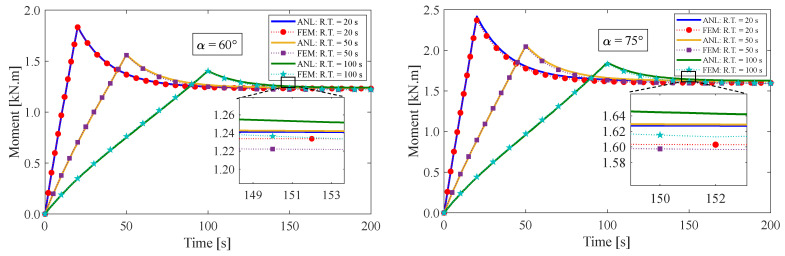
Variation of resultant bending moment of the bent bar for different fiber angles of α. The ultimate bending angle is defined as 180°.

Additionally, the impact of the fiber angle on the resultant bending moment is displayed in Figure 9. It has been observed that the maximum value of the moment rises with decreasing deformation rates. Based on the data presented in Figure 9, it is evident that the relaxed resultant bending moments maintain a consistent value at the ultimate bending angle, regardless of the rising time. These findings align with our expectations based on Equation (33), indicating that the resultant bending moment follows a similar trend as the tensile tangential stress.

### 4.2. Multi-Step Relaxation Test

**Figure 10:**
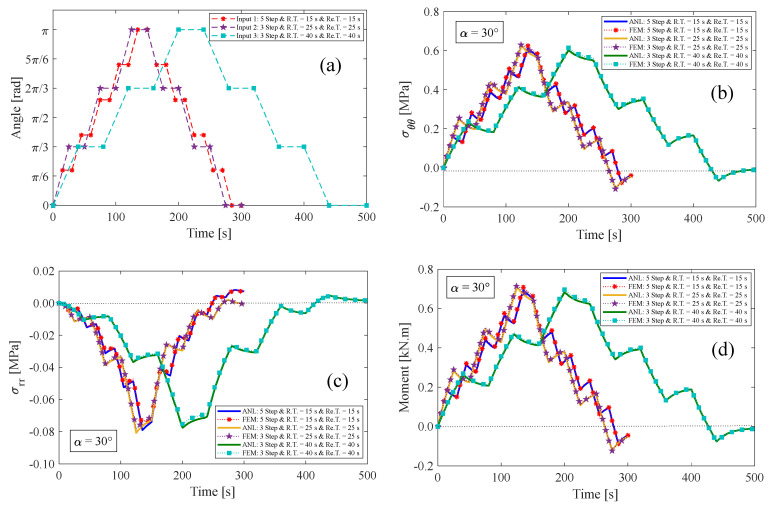
(**a**) Inputs of multi-step relaxation test, (**b**) tangential and (**c**) radial stress components, and (**d**) resultant bending moment for the fiber angle of α=30°.

**Figure 11:**
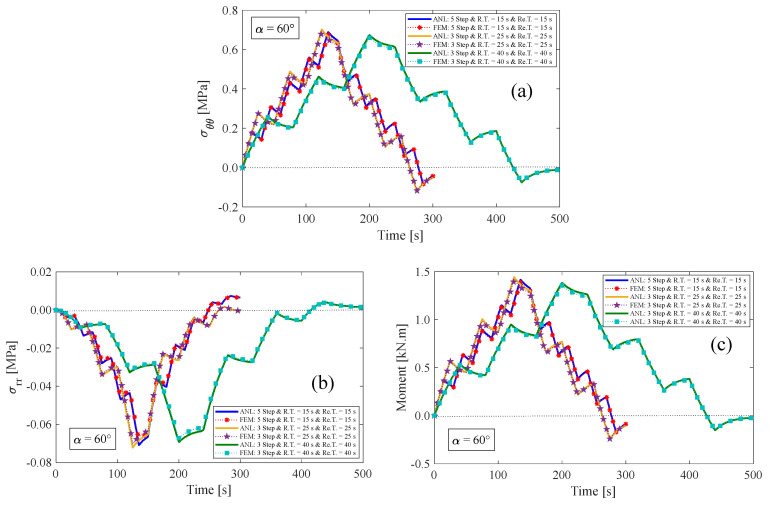
(**a**) Tangential and (**b**) radial stress components, and (**c**) resultant bending moment for the fiber angle of α=60°.

In this section, three multi-step relaxation tests are presented for an anisotropic visco-hyperelastic rectangular bar. The anisotropic bar is studied with two different initial fiber angles of 30° and 60°. Then, the results of these tests are compared with those obtained using the finite element (FE) method. During the loading condition, the bar undergoes bending from its original state up to a maximum bending angle of 180° in several steps. It is then returned to its initial unbent state in the opposite direction. The rising time (R.T.) and relaxation time (Re.T.) for each step are assumed to be equal. Figure 10a displays the inputs of multi-step tests, where the first input is composed of five bending stages and five relaxed stages. The second and third inputs, on the other hand, comprise three bending steps and three relaxed steps. It is worth mentioning that each stage of the aforementioned inputs has a duration of 15 s, 25 s, and 40 s, respectively. Figure 10 and Figure 11 illustrate the results of tangential stress on the outer surface, radial stress on the neutral axis, and the resultant bending moment of the anisotropic bar with fiber angles of 30° and 60°, respectively. It is evident that by returning the bar to its initial unbent state for the first time, some inverse residual stress persists in the material, which gradually vanishes over time. The obtained findings indicate a strong correlation between the presented analytical solution and the finite element methods.

## 5. Summary and Conclusions

As the bending of hyperelastic and viscoelastic materials is an important issue in engineering and medical industries, we attempted to study the bending of soft materials which are reinforced with fibers. As a result of this work, we will be able to gain a more comprehensive understanding of how soft materials behave under bending. In this research, a constitutive model was presented to analyze an incompressible anisotropic visco-hyperelastic rectangular bar under finite bending deformation. The proposed solution was expanded by employing the well-known anisotropic Mooney–Rivlin strain energy function for the strain-dependent part and non-linear viscoelastic method for the time-dependent part. A polymeric plane strain material reinforced by one family of fibers was investigated in this paper. The fibers were organized within the matrix in different directions. Moreover, a finite element analysis was carried out in order to verify the validity of the analytical formulation. To perceive the time-dependent behavior of the anisotropic rectangular bar, two well-known stress relaxation tests and multi-step stress relaxation tests were examined in the form of bending deformation. In the results section, the stress components, as well as the resultant bending moment, were investigated versus time. It was observed that the tangential stress on the outer radius of the bent anisotropic rectangular bar is tensile, and on the inner radius, it is compressive. Additionally, we concluded from the presented data that the amount of stress components changes with the change in the fibers’ angle. Furthermore, the effects of some variables such as rising time, ultimate bending angle, relaxation time, and the number of loading stages were taken into account. There was a high degree of conformity between the FEM results and the analytical results, which indicates the capability of the proposed constitutive model to study the mechanical behavior of anisotropic visco-hyperelastic materials under finite bending deformations. It was observed that at the same bending angle, the stress components and the resultant bending moment declined with increasing rising time, and also, their values increased substantially with the increasing curvature of the bar. It can be noted that in fiber-reinforced soft materials, the ultimate tensile and compressive strengths can differ depending on the structure, hardness, and direction of the embedded fibers.

## Figures and Tables

**Table 1:** Material properties calibrated from Yang, Yao, Yan, Yuan, Dong, and Liu [21] for polymeric matrix material and borrowed from Allahyari and Asgari [41] for reinforcing fibers.

Mooney–Rivlin Strain Energy Function Parameters			
$c_{1}(\mathrm{Pa})$	$c_{2}(Pa)$	$k_{1}(Pa)$	$k_{2}(-)$
315,000	17,200	150,000	0.2
**Viscoelastic parameters**			
$\beta_{i}(-)$		$\tau_{i}\;(s)$	
0.8		20	

**Table 2:** Maximum error between the analytical and numerical results for the fiber angles of $\alpha =\left[0{}^{\circ},\;90{}^{\circ}\right]$.

	Maximum Error (%)						
	α=0°	α=15°	α=30°	α=45°	α=60°	α=75°	α=90°
$\sigma_{\theta \theta }$ (Outer surface)	0.8	2.4	2.9	1.5	1.7	2.0	0.7
$\sigma_{\theta \theta }$ (Inner surface)	1.1	3.4	3.8	2.5	1.7	2.2	1.3
$\sigma_{zz}$ (Outer surface)	1.5	2.9	3.7	3.2	1.9	1.4	1.1
$\sigma_{zz}$ (Inner surface)	1.0	2.6	3.0	2.8	2.5	1.7	0.8
$\sigma_{rr}$ (Neutral axis)	0.9	2.0	2.4	0.6	5.6	5.2	1.9
$\sigma_{r\theta }$ (Outer surface)	0	2.1	3.0	0.3	3.6	2.9	0
$\sigma_{r\theta }$ (Inner surface)	0	1.8	2.6	0.4	3.2	2.8	0

## Data Availability

Data are contained within the article.
